# The role of inflammation in the development of epilepsy

**DOI:** 10.1186/s12974-018-1192-7

**Published:** 2018-05-15

**Authors:** Amna Rana, Alberto E. Musto

**Affiliations:** Department of Pathology and Anatomy, Department of Neurology, Eastern Virginia Medical School, 700 W. Olney Road, Lewis Hall, Office 2174, Norfolk, VA 23507 USA

**Keywords:** Epileptogenesis, Inflammation, Neurological disorders, Systemic inflammatory disorders, Blood–brain barrier (BBB) breakdown

## Abstract

Epilepsy, a neurological disease characterized by recurrent seizures, is often associated with a history of previous lesions in the nervous system. Impaired regulation of the activation and resolution of inflammatory cells and molecules in the injured neuronal tissue is a critical factor to the development of epilepsy. However, it is still unclear as to how that unbalanced regulation of inflammation contributes to epilepsy. Therefore, one of the goals in epilepsy research is to identify and elucidate the interconnected inflammatory pathways in systemic and neurological disorders that may further develop epilepsy progression. In this paper, inflammatory molecules, in neurological and systemic disorders (rheumatoid arthritis, Crohn’s, Type I Diabetes, etc.) that could contribute to epilepsy development, are reviewed.

Understanding the neurobiology of inflammation in epileptogenesis will contribute to the development of new biomarkers for better screening of patients at risk for epilepsy and new therapeutic targets for both prophylaxis and treatment of epilepsy.

## Background

Epilepsy is a multifaceted neurological disease, characterized by recurrent spontaneous seizures. Despite the efficacy of current anti-epileptic drugs, almost 30% of patients with epilepsy are refractory to medical treatment, have progressive cognitive impairment, and may require neurosurgical resection of the epileptic focus to ameliorate seizure recurrence [1].

It has been widely ascertained that the development of epilepsy-epileptogenesis can be owed to a diverse array of factors, including genetic predisposition, developmental dysfunction, and neurological insult, which contribute to morphological synaptic changes and hyper-excitable neuronal transmission [2]. For instance, a history of familial epilepsy, neurodevelopmental abnormality, and generation of complex febrile seizures are associated with the highest risk of epilepsy development in infants [2, 3]**.** Moreover, neurological insult, such as traumatic brain injury, hypoxia, or febrile seizures, is associated with neuronal death, dysfunctional synaptic modification, and the generation of a hyper-excitable network, which could predispose to spontaneous recurrent seizures [1]**.** Brain injury induces a highly regulated cascade of biological events, characterized by the release of cytokines, chemokines, lipid mediators, and protectins in the neuronal microenvironment [4, 5]**.** Physiologically, inflammatory mediators activate their corresponding receptors on different brain cells to stimulate various pathways of molecular signaling and to initiate brain repair [6]. Deregulation of mediator and receptor expression could sustain neuronal damage, which would clinically manifest depending on the region of the brain affected [7]**.** Although the cellular and molecular mechanisms of epileptogenesis are not clear, it is postulated that focal or systemic unregulated inflammatory processes lead to aberrant neural connectivity and the hyper-excitable neuronal network, which mediate the onset of epilepsy [4, 8]**.**

**Fig. 1:**
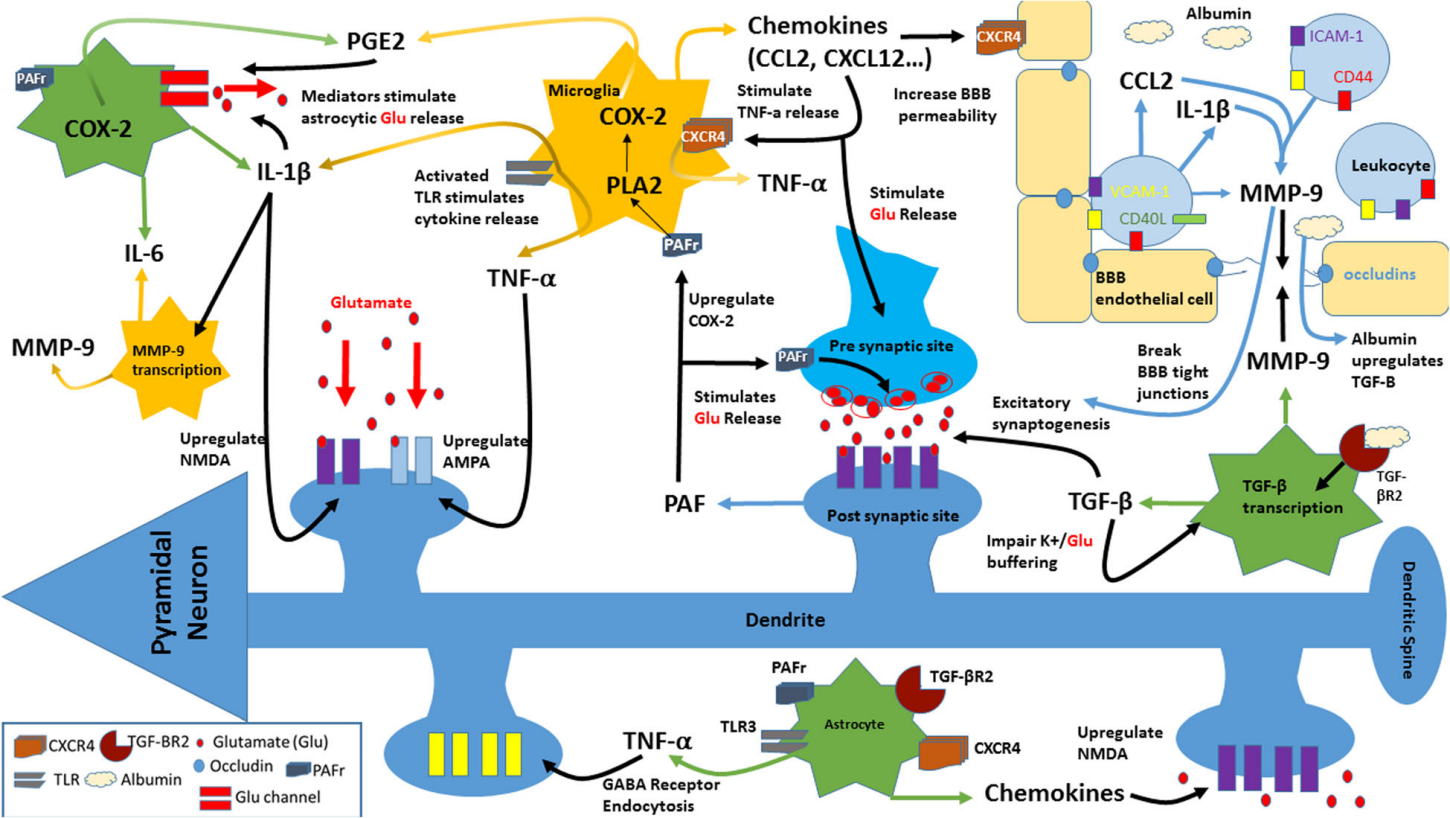
Sources and targets of unregulated and overlapped components of inflammation in epileptogenesis. Brain injury induces central inflammation and aberrant neuronal connectivity within the hippocampus. Systemic inflammatory disorders generate peripheral inflammation which can further contribute to the buildup of inflammatory mediators. Peripheral and central inflammation allow for the breakdown of the blood–brain barrier due to the upregulation of inflammatory mediators. BBB breakdown permits leukocyte infiltration which generates neuronal hyper-excitability and further upregulates inflammatory mediators. Unregulated peripheral and central inflammation and breakdown of the blood–brain barrier lead to morphological synaptic changes within the hippocampus and ultimately, the development of epilepsy

This review discusses critical inflammatory events, from neuronal tissue (central inflammation), BBB integrity and systemic inflammatory disorders (peripheral inflammation), that could contribute to epilepsy and may hold potential as molecular biomarkers and targets for therapeutic approaches for epilepsy (Fig. 1).

## Inflammation in the central nervous system

Epileptogenesis is associated, along with subtle neuronal damage, gliosis, and microgliosis, with an increased, strong, and persistent inflammatory state in the microenvironment of neural tissue [9]. Inflammatory processes may originate in the central nervous system or be acquired from systemic circulation through a breakdown in the blood–brain barrier (BBB) [10]. A wider breadth of research on neuro-inflammation and epilepsy has focused on hippocampal foci as opposed to extra-hippocampal foci of epilepsy due to the well-documented pathology, physiology, and clinical manifestations of hippocampal atrophy and sclerosis. However, evidence has also associated neuro-inflammation with extra-hippocampal neuronal cell death and gliosis [11]. Various clinical trials with TSPO (translocator protein expressed by activated microglia) positron emission tomography (PET) have linked seizures, induced in temporal lobe epilepsy, frontal lobe epilepsy, and focal cortical dysplasia, with ensuing acute neuro-inflammation. The acute bout of neuro-inflammation is thought to contribute to and worsen a pre-existing state of chronic neuro-inflammation [12]. For instance, patients with multiple sclerosis (MS) have increased risk for epilepsy development when MS is associated with the development of pure intracortical lesions, which cause extensive cortical inflammation [13]. A deeper understanding of the chemical mediators and receptors relevant to neuro-inflammation may elucidate their neurobiological mechanistic contribution to epileptogenesis.

### Cytokines

Cytokines, proteins that modulate inflammatory processes, are primarily produced by glial cells and neurons during brain inflammation [9]. Pro-inflammatory cytokines, interleukin-1β (IL-Iβ), IL-2, and IL-6, typically concentrated in low quantities within the brain, increase after seizures [14]. In a clinical study, febrile seizures increased levels of cytokines IL-1β, IL-6, and tumor necrosis factor- α (TNF-α) in cerebrospinal fluid [15]. In addition, mRNA expression of cytokines IL-1β, IL-6, and TNF-α, along with transforming growth factor- beta 1 (TGF-β1) and vascular endothelial growth factor (VEGF), is upregulated in the hippocampus following seizures [14, 16, 17]. Such key cytokines and cytokine receptors implicate various mechanistic inflammatory pathways which cause detrimental synaptic changes and neuronal hyper-excitability.

### Interleukin-1β

Pro-inflammatory cytokine IL-Iβ, expressed in activated microglia and astrocytes, enhances the release of glutamate from astrocytes and decreases glutamate re-uptake, thereby increasing glutamate availability in neuronal synapses and promoting neuronal hyper-excitability [9]. It has been suggested that IL-1β induces seizures through the upregulation of NMDA receptors on post-synaptic cells via an activation of the GluN2B subunit of the NMDA receptor [18]. Postnikova et al., using models of epilepsy, found that the production of the GluN2B mRNA increases 24 h after seizures and that changes in NMDA receptors may lead to impaired synaptic plasticity [19]. It has also been reported that induced seizures decreased long-term potentiation (LTP), a physiological marker of synaptic strength via normal plasticity, while IL-1β antagonist anakinra increased LTP. These findings suggest that unregulated levels of IL-1β impair physiologic synaptic plasticity and may cause potential neuronal dysfunction [16]. Another study by Roseti et al. shows that the pathophysiological concentrations of IL-1β in TLE decrease GABA-mediated neurotransmission by up to 30% and lead to seizure generation due to neuronal hyper-excitability [20]. Cytokine IL-1β has also been found to be significantly increased within the cerebrospinal fluid (CSF) in the epileptic pediatric population as opposed to the control group, suggesting the cytokine’s important role in epilepsy initiation and progression [21].

### Tumor necrosis factor-α

Pro-inflammatory cytokine TNF-α is released from activated microglia and astrocytes. Glial cells sense extracellular glutamate levels and, upon detection of low levels of glutamate, release TNF-α to upregulate synapses and maintain a certain level of neuronal excitatory input [22]. It has been reported that TNF-α regulates N-cadherin, an adhesion molecule involved in the formation and organization of excitatory and inhibitory synapses [23]. Furthermore, TNF-α has been found to increase microglial glutamate release through the upregulation of glutaminase and gap junctions in microglia [24]. In addition, TNF-α upregulates AMPA receptors, augmenting glutamergic transmission. Increased AMPA receptors allow for the over-uptake of calcium, causing neurotoxicity [25]. TNF-α not only amplifies the number of glutamate receptors but also induces GABA receptor endocytosis, reducing the inhibitory drive and causing pertinent changes in excitability [26]. Although TNF-α may have an important role in epileptogenesis, anti-TNF-α therapy for epilepsy is under debate due to the suspected risks of infection and cancer development. Within the TNF and TNF receptor (TNFR) family, certain ligands have come to light as possible pharmacological targets. In patients with TLE, TNF-related apoptosis-inducing ligand (TRAIL) expression is increased to significant levels and is thought to modulate chemokine (C-X3-C motif) ligand 1 (CX3CL1)-induced cell death [9, 27]. There may be an association with hippocampal sclerosis but the effects of TRAIL and TRAIL receptors must be further analyzed to be fully understood [9].

### Interleukin-6

IL-6, a pro-inflammatory cytokine, is typically found in low quantities in the central nervous system, but stimulation of astrocytes and microglia can lead to increased production of IL-6 [28]. IL-6 is also upregulated by increased levels of other cytokines such as TNF-α, IL-Iβ, IFN-gamma, and IL-17 [29]. Studies indicate that IL-6 upregulation decreases LTP and hippocampal neurogenesis while increasing gliosis, creating conditions that may contribute to epileptogenesis [29, 30]. Although in one study IL-6 knockout (KO) mice were found to be more sensitive to pro-convulsant stimuli, in other studies, transgenic IL-6 expression and intranasal IL-6 administration within mice proved to be pro-convulsive [29]. IL-6 has also been found to bi-directionally transfer across human placenta [31, 32]. It is suggested that prenatal exposure to IL-6 results in higher risk of neurodegeneration of the hippocampus, leading to changes in both hippocampal structure and morphology [33]. Polyinosinic–polycytidylic acid (PIC) induces maternal immune activation (MIA) in experimental pregnancy, leading to hippocampal hyper excitability and faster progression of epileptogenesis in the offspring by increasing pro-inflammatory cytokines IL-6 and IL-1β within the offspring hippocampus [34]. These findings further implicate the role of IL-6 in epileptogenesis. However, although it is clear that IL-6 contributes to neural inflammation-induced epilepsy, more research is required for IL-6 to be considered in disease-modifying therapy.

### Prostaglandins

Prostaglandins (PG) are formed from arachidonic acid by the constitutively expressed cyclooxygenase-1 (COX-1) and the inducible COX-2 enzymes and are secreted mainly by astrocytes and microglia. Prostaglandin E2 (PGE2) is coupled with its receptors EP1, EP2, EP3, and EP4 [35–37]. PGE2 stimulates EP3 on astrocytes, increasing astrocytic glutamate release and inducing hyper excitability and neuronal cell death; meanwhile, inhibition of EP3 may delay seizure induction [35]. mPGES (membrane-bound PGE2 synthase) increases production of glial fibrillary acidic protein (GFAP)-positive astrocytes following seizure kindling, whereas PGE2 antagonists reduce seizure severity and seizure-induced neurological damage in experimental epilepsy [35, 38]. Although PGE2 synthesis may be an effective therapeutic target, the role of COX-2 as a therapeutic target remains unclear. Studies show that COX-2 deficiency in immature mouse brains leads to greater seizure susceptibility due to decreased production of anticonvulsants PGF-2a and PGD-2 [39, 40]; PGD-2 is reported as essential to seizure suppression [41]. However, in a recent study conducted by Iwasa et al., the blockade of PG production ameliorated delayed neuronal death mediated by PGD-2 [42]. Current evidence is inconclusive on the effectivity of COX-2 therapy, and many have reported that the effects of COX-2 therapy may be highly dependent on the type of inhibitor used and the timing at which the medication is administered in relation to seizure onset. Such conflicting results are drawing attention to other targets within PG synthesis and mechanistic pathways [43]. COX-2 is upregulated by platelet-activating factor (PAF) and NMDA activation [7, 44].

### Platelet-activating factor

PAF, a potent pro-inflammatory lipid mediator, has both physiological and pathological implications in the brain. PAF stimulates glutamate release and is a retrograde-messenger for LTP [45]. PAF also activates transcriptional signaling pathways of COX-2 gene expression [45, 46]. The action of PAF is mediated through its interaction with distinct binding sites on presynaptic and intracellular membranes [44]. PAF binding sites have been successfully blocked by PAF receptor antagonists, which are neuroprotective in brain damage and downregulate cytokine production and COX-2 induction in human neuronal cells [47–49]. Epileptogenesis and aberrant plasticity associated with epilepsy are attenuated by PAF receptor antagonists [7, 50].

### CD44

Adhesion molecules such as CD44 are also implicated in epileptogenesis. CD44 is physiologically expressed on glial cells and neurons and acts as a signaling molecule that guides neuron development [51]. In response to stress such as glutamate mediated neuronal hyper excitability, CD44 has been found to induce Src kinase activation, leading to actin remodeling and alterations of dendritic morphology such as hippocampal dendritic shortening [51]. Changes in dendritic morphology, though under debate, have been shown to contribute to epileptogenesis [52]. CD44 is also upregulated in epileptogenesis and is thought to play a role in neuronal reorganization and mossy fiber sprouting (MFS), a process of hippocampal synaptic rearrangement [53]. In addition, the CD44 ligand, hyaluron, has been found to have a 146% increase in the hippocampus of patients with mesial TLE and the upregulation of hyaluron has been implicated in mossy fiber sprouting [54–56]. CD44 silencing decreased dendritic morphology changes, which suggests CD44 silencing to be a therapeutic mechanism for epileptogenesis [51].

### Matrix metalloproteinase-9

Matrix metalloproteinase-9 (MMP-9) is a proteolytic enzyme secreted primarily by astrocytes and microglia in the hippocampus, cerebral cortex, and cerebellum. MMP-9 transcription increases in response to depolarization of neurons and upregulation of other inflammatory factors such as IL-Iβ and chemokines. MMP-9 has various roles within the brain, ranging from structural modifications to facilitation of inflammatory processes. Chronic levels of MMP-9 cause thinning and elongation of dendritic spines, leading to morphological changes in synapses, and are associated with impaired synaptic plasticity [57–59]. Moreover, increased expression of MMP-9 indicates greater susceptibility to epileptogenesis as MMP-9 facilitates cell death through various mechanisms including excitotoxicity, apoptosis, and impairment of extracellular matrix-cell interactions [57, 58]. MMP-9 also loosens the BBB by directly damaging the tight junctions. Furthermore, MMP-9 activity increases in neural and glial cells, within the CA1 and CA2 regions of the hippocampus in patients with mesial TLE (MTLE) with hippocampal sclerosis, and is implicated as an important factor in drug resistant MTLE, suggesting a potential therapeutic target in patients with drug-resistant epilepsy [58].

### Toll-like receptors

Toll-like receptors (TLR 1, 2, 3), expressed primarily by microglia and astrocytes, mediate innate and adaptive immunity. TLRs induce secretion of cytokines such as IL-1β and other inflammatory mediators which mediate epileptogenesis [60]. In an experimental model of epilepsy, deletion of TLR3 limits seizures, reduces levels of cytokines TNF-α and IL-1β, decreases levels of microglial activity, and increases survival rates [61]. TLR3 contributes to hippocampal excitability through its upregulation of pro-inflammatory cytokines such as IFN- β [62]. FOX3P, typically found to induce T cell differentiation, is also expressed by microglia to downregulate inflammatory processes through modulation of NFkB, a key inflammatory transcription factor [63]. FOX3P limits TLR4 signaling and inflammation, leading to an inactivation of the NR2B NMDA receptor and attenuation of seizure activity [64]. This research suggests TLRs as possible therapeutic targets due to their intrinsic importance in neuro-inflammation and potential restructuration of neuronal excitability.

### Chemokines

Chemokines, expressed in the brain by microglia, astrocytes, and endothelial cells, guide inflammatory mediators towards the source of inflammation and activate leukocytes [65, 66]. Many variants of chemokines can alter neuronal physiology through the modulation of voltage-dependent channels, activation of G-protein-gated potassium influx channels, and increased release of certain neurotransmitters [66]. Chemokines CCL2, CCL3, and CCL4 have been detected in DNA microarray analysis of surgically removed hippocampi of TLE patients [66]. TLE patients also exhibit increased C-X-C chemokine receptor type 4 (CXCR4) expression on microglia and astrocytes, which leads to an increase in CXCL12 binding, stimulating microglia to release TNF-α and then increasing glutamate levels [66]. Chemokine CCL2, which binds to the G-protein-coupled receptor C-C chemokine receptor type 2 (CCR2), is also highly elevated in patients with pharmacoresistant epilepsy and is found on various brain cell types, including neurons, astrocytes, microglia, neural progenitor cells, and microvascular endothelial cells, suggesting that CCL2 and its receptor CCR2 could play an important role in seizure control [65]. Moreover, in patients with intractable epilepsy and in experimental epilepsy models, CXCL13 and CXCR5 are highly upregulated, suggesting a mediation in epileptogenesis [67].

### Neurological disorders and epilepsy

Insult to the brain typically induces an acute neuro-inflammatory response, marked by an increase of pro-inflammatory molecules, which mediate onset of recurrent seizure development [4, 8]. Neural inflammation initiated due to other neurological pathologies (see Table 1) may contribute to the development of epilepsy as inflammatory mediators are upregulated. As a result, the treatment and resolution of other neurological conditions may play a key role in the prevention of epilepsy.

**Table 1:** Neural inflammation initiated due to other neurological pathologiesᅟ

Neurological disorders	Inflammatory contribution to epilepsy development
Traumatic brain injury	Damage associated molecular pattern (DAMP) stimulation of immune system Significant CCL2 increase Neutrophil recruitment —> BBB damage Reactive oxygen species (ROS) release —> activate vascular endothelium —> T cell infiltration of BBB [137]
Status epilepticus	Elevated IL-1β, TNF-α, IL-6 transcript levels Albumin extravasation <—>BBB breakdown EP2-COX2 upregulation TLR pathways stimulated [138]
Multiple sclerosis	Increased oxidative stress and ROS Activation of microglia Recruitment of T-cells, B-cells, and macrophages [139]
GBM	IL-8 up-regulation via EGFRvIII IL-6-mediated STAT3 activation IL-1β-dependent activation of NF-κB, p38 MAPK and JNKs pathways [140]
Stroke	TNF-α, IL-1Β, IL-6 upregulation ICAM and VCAM upregulation MMP-9 increase MCP-1,(MIP-1α), fractalkine (CX3CL1) increase [141]
Alzheimer’s disease	MCP-1, cytokines (IL-6, TNF-α), CXCL8, CCL5 increase Deposition of Aβ generates ROS Activation of the complement cascade [142]

## Blood–brain barrier breakdown

The CNS is generally protected against many of the conventional reactions present in the immune system due to the BBB, which is impermeable to many molecules, toxins, and cells due to numerous non-infenestrated endothelial cells with inter-endothelial tight junctions. This delicate layer is maintained by the normal function of pericytes, perivascular microglia, astrocytes, and the basal lamina [10]. Physiologic astrocyte production of the sonic hedgehog protein signals endothelial cells to secrete Netrin 1, a laminin like protein which regulates glial cell migration. Netrin 1 increases endothelial junctional proteins to stabilize the BBB and decreases leukocyte infiltration, suggesting an important role as a BBB protective mediator [68].

### Inflammatory mediators overview

Central and peripheral inflammation contributes to the breakdown of the BBB through the upregulation of inflammatory mediators. SE, infections, and traumatic and ischemic injuries have been reported to cause transient changes in the composition and permeability of the BBB. Although the exact mechanism of delayed onset of epilepsy remains unclear, available data suggests that inflammation and breakdown of the BBB are necessary components of epileptogenesis following brain injury [10]. For instance, gram-negative bacterial lipopolysaccharide (LPS) activates macrophages to produce IL-1, IL-6, and TNF alpha, promoting BBB permeability and facilitating entrance of peripherally generated pro-inflammatory cytokines into circumventricular brain regions [69]. TNF-α and IL-6, cytokines that increase the BBB permeability, have been clearly implicated in seizure generation and severity [70–72]. IL-1β is also an etiologic trigger for BBB breakdown and plays a pivotal role in the activation of astrocytes whereas IL-1Ra, an IL-1 antagonist, limits the effects of those cytokines [10]. In addition, the binding of cytokines to receptors located in brain vasculature can cause the production of molecules, such as endothelial cell adhesion molecules, chemokines, nitric oxide, and prostaglandins that may further compromise the integrity of the BBB [69].

### Leukocyte adhesion and infiltration

BBB leukocyte-endothelium interactions mediate leakage and infiltration of inflammatory cells into the hippocampus [73]. Epilepsy is typically accompanied by an increase of leukocytes, such as neutrophils, into the hippocampus, and the infiltration is thought to lead to higher levels of neurodegeneration [73, 74]. Spontaneous recurrent seizures lead to the chronic expression of VCAM-1, the ligand for VLA-4 integrin. It has been hypothesized that upregulation of VCAM-1 may contribute to BBB permeability, neuro-inflammation, and subsequent seizure generation [73]. CD44 mediates, in conjunction with the integrin pathway, leukocyte adhesion and rolling on cytokine-activated endothelium [75, 76]. Using induced CD44, VCAM-1, and ICAM-1, leukocytes form endothelial membrane protrusions, called “transmigratory cups”, which assist with trans-endothelial migration of leukocytes across the BBB [76]. Of particular note, one study suggests that CD44 is required for optimal neutrophil recruitment into tissues as CD44 (−/−) mice indicated a 65% decrease in neutrophil adhesion [77]. Certainly, leukocyte-adhesion molecule targets may be looked into for epilepsy treatment [74]. However, genetic and pharmacological interventions to prevent T-cell infiltration or deplete systemic macrophages and cytotoxic inflammatory cells show diverse outcomes, suggesting that the mechanics of neuro-inflammatory signaling in epileptogenesis are incompletely understood [74, 78].

Leukocyte infiltration through dysfunctional BBB leads to the upregulation of inflammatory mediators, such as interleukins, tumor necrosis factors, COX-2, complement, and adhesion molecules which contribute to leakage of the BBB. Specifically, findings show that T cell activation leads to the release of chemokines. CCL2 mediates chemotaxis of neutrophils and enhances local inflammatory responses in the brain [79–82]. Inflowing leukocytes also secrete MMP-9, which then damages the BBB by cleaving the zonula occludens 1 protein, which is integral to the tight junctions of the BBB. By directly damaging the BBB, MMP-9 allows for greater inflow of leukocytes and perpetuates the inflammation. MMP-9 also cleaves dystroglycan, a protein which fixes astrocytic feet to the basement membrane and allows for leukocytic infiltration into the brain’s parenchyma [57]. In addition, CD40-L is expressed predominantly on activated CD4+ T lymphocytes and increases after SE [83]. The CD40 Ligand (CD40-L) is a transmembrane glycoprotein belonging to the TNF family, and the soluble trimeric form of CD40-L has the most potent biological activity through oligomerization of cell surface receptor, CD40 [84]. The CD40-CD40L pathways have been implicated in many neurological disorders; however, the research related to epilepsy is sparse. Further research into this pathway may yield important molecular targets.

### Astrocyte reactivity

Evidence suggests that the BBB disruption contributes to astrocyte activation and gliosis. Within 24–48 h of the induction of SE, there is activation of glial fibrillary acidic protein-positive astrocytes (GFAP). The reactive gliosis may be detectable for over 3 to 4 months [10]. These reactive astrocytes may have difficulty handling the extracellular glutamate, leading to neuronal hyper excitability and damage. Reactive astrocytes also serve to further release pro-inflammatory cytokines (e.g., IL-1β, IL-6, and TNF-α) and recruit more inflammatory cells by secreting C-C motif chemokine ligands 2, 3, and 5 [57]. As a result, there is increased neuronal excitability, development of seizures, cell death, and subsequent neuro-inflammation [72]. The leakage of serum proteins such as albumin through a disrupted BBB may be a key factor in the initiation of specific signaling cascades within neurovascular cells, specifically astrocytes. Albumin is hypothesized to bind to the astrocytic TGF- βR2, leading to the activation of the TGF-β signaling pathway, production of TGF-β, and astrocyte activation, which causes impaired buffering of potassium and glutamate at the cellular level [85]. ALK5/TGF-β-pathway induces excitatory synaptogenesis whereas SJN2511, a specific ALK5/TGF-β inhibitor, prevents synaptogenesis and epilepsy [86]. Hence, TGF-β pathway inhibition prevents the activation of astrocytes during epileptogenesis, leading to a reduction in spontaneous seizure activity and brain inflammation [85, 87]. The TGF-Β pathway may serve as a therapeutic target to prevent seizure development in individuals with brain injury [86].

## Systemic inflammation

As discussed, inflammatory molecules are induced in response to brain injury, such as SE, and modulate not only neural connectivity and excitability but contribute to the breakdown of the BBB, allowing for further intrusion of harmful chemicals and mediators. Various experimental models have shown that the systemic administration of LPS leads to the generation of seizures and enhancement of epileptogenesis. LPS-induced peripheral inflammation increases seizure susceptibility through COX-2-dependent microglial activation and upregulation of IL-1β, IL-6, and TNF-α in the hippocampus [88]. Co-administration of LPS and IL-1B receptor antagonist in immature rat brains, however, partially reverses the enhancement of epileptogenesis [89]. Furthermore, the systematic disruption of CCL2 signaling, through intracerebral administration of anti-CCL2 antibodies, results in potent suppression of LPS-induced seizures in chronically epileptic animals [90]. CCL2 is suggested to be a key mediator that bridges peripheral inflammation and epilepsy. These research findings suggest an important association between peripheral inflammatory conditions and epilepsy.

Furthermore, in a population based retrospective cohort study (*n* = 2,518,034), the relationship between autoimmune diseases, such as SLE, Hashimoto’s Thyroiditis, and RA, and epilepsy was examined. It was found that all of the autoimmune disorders examined posed a varying increased risk of epilepsy and, as a whole, autoimmune diseases posed a fivefold increased risk of epilepsy in children and fourfold increased risk of epilepsy in non-elderly adults (age < 65) [91]. An extensive meta-analysis study investigated the relationship between systemic autoimmune disorders (SAD) and epilepsy and found a 2.5-fold increase of epilepsy in SAD, 2.5-fold increase of SAD in epilepsy, and established a stronger association of SAD and epilepsy in individuals younger than 20 [92].

Due to the evident statistical association of epilepsy and systemic inflammatory conditions, combined with the biochemical understanding of peripheral inflammation leading to central inflammation due to the breakdown of the blood–brain barrier, it is imperative to further study the relationship between systemic inflammatory conditions and epilepsy for prevention and treatment. Most mechanistic pathways between these inflammatory conditions and epilepsy remain un-elucidated. The following sections present the current understanding of the relationship between epilepsy and inflammatory conditions, as well as note possible contributory inflammatory factors (Table 2).

**Table 2:** Inflammatory conditions

Systemic inflammatory condition	Associated inflammatory findings	Odds ratio (compared to control group)	Prevalence of epilepsy	Incidence of epilepsy
Systemic lupus erythematous (SLE)	Antibodies: ANA, dsDNA, SS-A/SS-B.Upregulates anti- MAP-2 and anti NMDR presence in CSF IL-1β, Il-8, IFN gamma activate microglia. IL-10 upregulates spinogenesis	7-fold [91]	2.5% [91] Up to 8 times higher than non-SLE population [92, 93]	2.86-fold greater than non-SLE cohort [96]
Rheumatoid arthritis (RA)	Upregulated TNF-α,IL-1 and IL-6 cytokines	3.5-fold [91]	1.2% [91]	1.27-fold greater than control cohort [130]
Type I diabetes	Autoantibodies GAD 45	5.2-fold [91]	1.8% [91] 2–3.7% [120]	2.2-fold greater than control cohort [143]
Celiac disease	Anti-endomysial antibodies (EMA), anti-tissue transglutaminase antibodies (tTG), and anti-gliadin antibodies (AGA)	4.5-fold [91]	1.5% [91]	–
Sjogrens	Lymphocyte infiltration of CD4+ T cells, B cells, and plasma cells	4.3-fold [91]	1.5% [91]	1.5-fold greater than control [122, 124, 125]
Crohn’s Disease	Th1 and Th17 pathways CD44, IL-6, TNF-a upregulated	3.1-fold [91]	1.1% [91] 3.5–5.9% [105]	–
Ulcerative Colitis	IL-5, IL-13, IL-15 with Th2 upregulation	2.5 fold [91]	0.9% [91]	–
Hashimoto’s thyroiditis	Circulating immune complexes Anti-thyroid antibodies	2.4-fold [91]	0.8% [91]	–
Behcet’s	HLA-B51 Neuronal lymphocytosis	–	2.2–5% [105, 108]	–
Anti-phospholipid syndrome	Antibodies directed against membrane anionic phospholipids	3.2-fold [94, 103, 119]	–	–

### Systemic inflammatory conditions

#### Systemic lupus erythematous

Systemic lupus erythematous (SLE) is a chronic autoimmune disease characterized by an increase of anti-nuclear and anti-glomerular autoantibodies as well as immunological events that affect several organs through the activation of innate and adaptive immunity [93]. The prevalence of epilepsy in SLE is up to eight times higher than that in the general population, and seizures may appear many years before SLE is diagnosed. Some SLE cases are associated with anti-phospholipid and anti-cardiolipin antibodies and are correlated with abnormal MRI findings in the brain [10, 94, 95]. Vasculitis-stroke-like events could trigger seizures in SLE as a consequence of coagulopathy in small brain vessels or circulation of anti-neuronal complexes that invade the brain tissue [96]. In that case, epileptic seizures could indicate repetitive acute immune events that impact a particular neuronal network. SLE may also induce abnormal plasticity and promote aberrant neuronal connections that are responsible for seizures and eventual development of epilepsy. IL-10 is associated with super antigen polymorphism and SLE [97]. In the presence of BBB damage after SLE vasculitis, IL-10 crosses the BBB and induces spinogenesis, increasing excitatory and inhibitory synaptic contacts [98]. SLE also increases prolactin which modulates neuronal activity [99]. SLE up regulates anti- MAP-2 and anti NMDR presence in CSF and upregulates circulating IL-1β, Il-8, IFN gamma, and antibodies which activate microglia [97, 100–102]. Moreover, increased levels of anti-phospholipid antibodies are associated with increased seizure development [103].

#### Hashimoto’s encephalopathy

Hashimoto’s encephalopathy (HE), a rare complication of autoimmune thyroiditis, involves a high anti-thyroid antibody titer, and clinical manifestations include seizures, agitation, and cognitive deterioration [104]. Up to 66% of HE patients present with seizures, and both convulsive and non-convulsive forms of status epilepticus have been reported in patients with HE. Furthermore, in one study, 80% of HE patients presented with elevated protein and lymphocytic pleocytosis. Specifically, the pathophysiology of HE may involve circulating immune complexes or neuronal antibodies [105]. HE patients respond dramatically to corticosteroids, and this response suggests an immune mediated pro-inflammatory reaction as a possible cause of the CNS symptoms [106]. Although thyroid hormones mediate neuronal activity, it is difficult to separate how much involvement of thyroid hormones and neuro-inflammation exists and how their relationship upregulates the neuronal network that could mediate recurrent seizures in epilepsy [104].

#### Behcet’s disease

Behcet’s disease (BD), characterized by recurrent oral ulcers, genital ulcers, and inflammation of the middle layer of the eye, is associated with epilepsy [107]. BD is common in individuals of Middle Eastern and Central Asian descent and those with HLA-B51. Amongst these individuals, seizures and epilepsy occur in 2.2–5% of the cases. Most seizures are of the tonic–clonic type, but partial seizures, like epilepsia partialis continua, are also associated with BD [105, 108]. In Caucasian patients with neuro-Behcet’s disease, 27% suffered either single or recurrent seizures and 50% of patients had pleocytosis in their CSF [109]. Seizures in BD have shown improvement with immunosuppressant therapy [108].

#### Type I diabetes

Type I diabetes (T1DM) is a condition associated with an increase of autoantibodies GAD 45 [110, 111]. These antibodies are present in the CSF of T1DM patients [112–115]. Up to 2–3.7% of patients with T1DM have epilepsy, and patients with type I diabetes have up to six times the increased risk for epilepsy [116].

#### Crohn’s disease

Crohn’s disease is a chronic inflammatory disorder characterized by the upregulation of pro-inflammatory cytokines and induction of Th1 and Th17 pathways [117]. According to one study, epilepsy occurs in 3.5 to 5.9% of Crohn’s patients, while general neurological manifestations occur in 33 to 67% of Crohn’s patients [105]. Crohn’s disease is specifically associated with the upregulation of the NF-kB transcription factor with high levels of TNF-α and IL-6 [70–72]. CD44 molecules also double on peripheral blood cells and lymph nodes in Crohn’s disease [118]. Further clinical and biomedical research is required to better understand the relationship between Crohn’s and epilepsy.

#### Antiphospholipid syndrome

Antiphospholipid syndrome, a recurrent state of venous or arterial thrombosis, is associated with antibodies directed against membrane anionic phospholipids (i.e., anticardiolipin [aCL] antibody, antiphosphatidylserine) or corresponding plasma proteins, predominantly beta-2 glycoprotein I (apolipoprotein H). Patients with this condition have a 3.2-fold increased risk of developing epilepsy [94, 102, 119].

#### Sjogren’s syndrome

Sjogren’s syndrome (SS) is a chronic autoimmune disorder characterized by the lymphocyte infiltration of CD4+ T cells, B cells, and plasma cells in exocrine glands, mainly in salivary and ophthalmic glands. The disorder is also associated with involvement in other organs, including the brain. Although its cause remains unknown, the pathophysiology can be explained by interactions of several molecular factors [120]. Furthermore, between 2 and 60% of patients with SS present neurological complications [121]. SS patients with HDL-c particularly show white matter anomalies [122]. Reports show 1.5% incidence of epilepsy in SS patients and that 1/3 of SS patients have EEG abnormalities [123–125]. However, there is a lack of studies that have explored the relation of SS and epilepsy.

#### Ulcerative colitis

Ulcerative colitis (UC) is a chronic inflammatory bowel disease of unknown etiology characterized by ulceration of the colon. UC is thought to have extra-intestinal neurological manifestations associated with the peripheral nervous system [126].

#### Rheumatoid arthritis

Rheumatoid arthritis (RA) is an autoimmune inflammatory disorder characterized by the anti-IgG antibody (RF Factor) and anti-CCP antibody. It is associated with an upregulation of TNF-α, IL-1 and IL-6 [127–129]. The development of epilepsy within RA patients is 1.27-fold higher than the control patients without RA. Duration of non-steroidal anti-inflammatory drug (NSAID) therapy in RA patients negatively correlated with epilepsy development while patients with minimal NSAID therapy had a greater risk of epilepsy development [130]. This suggests that limiting the inflammatory process induced by RA reduces risk of epileptogenesis. Offspring of women, who had RA during pregnancy, are at a significantly higher risk of developing childhood-onset epilepsy as compared to the offspring of women who developed RA post-pregnancy. Paternal RA is suggested to have a relatively low association to epilepsy development within offspring. These findings suggest that the inflammatory conditions induced within RA impact the intrauterine environment and increase the risk of epileptogenesis within children [131].

#### Celiac disease

Celiac disease (CD) is an autoimmune disorder characterized by gluten sensitivity, predisposing factors such as HLA DQ2/8, and different antibodies including anti-endomysial antibodies (EMA), anti-tissue transglutaminase antibodies (tTG), and anti-gliadin antibodies (AGA) [132, 133]. Exposure to gluten induces an immune reaction which leads to villous atrophy, crypt hyperplasia, and increased intraepithelial lymphocytes [133]. Per a meta-analysis on epilepsy and systemic autoimmune disorders, patients with epilepsy have a 2.6-fold increased risk for celiac disease [92]. In an epidemiologic study of nearly 29,000 subjects with CD and 143,000 controls, it was found that CD increased risk of epilepsy by 1.4-fold [103]. Many case studies have reported an association between CD and epilepsy with occipital calcifications and cerebellar degeneration [133–135]. Another study established an association between TLE, hippocampal sclerosis, and celiac disease [135]. Furthermore, the implementation of a gluten-free diet in epileptic patients may contribute to a decrease of antiepileptic medication use. On a clinical level, CD screening may be advisable as a gluten free diet could possibly replace the need for medication and reduce the occurrence of seizures [136].

#### Clinical importance

Considering the aforementioned neurobiological effects of inflammatory mediators, the presence of one of these systemic inflammatory conditions could mediate development of epilepsy by (a) inducing seizures, which then beget further seizures and lead to onset of recurrent seizures and epileptogenesis; (b) altering neuronal network architecture which promotes spontaneous recurrent seizures; and (c) aggravating the course of epilepsy (secondary epileptogenesis). Therefore, it can be suggested that inflammation can be treated as an important factor in the treatment of patients with epilepsy. Certainly, prophylactic anti-inflammatory measures in patients at risk for epilepsy may serve to ameliorate the neuronal damage. Proper treatment and possible resolution of other systemic inflammatory disorders may also play a key role in suppressing epilepsy progression.

## Conclusion

The development of epilepsy is characterized by complex unregulated inflammatory molecules and pathways found in both the nervous system and systemic tissue. Understanding the neurobiology of cytokines, chemokines, MMP-9, and adhesion molecules, overlapping with neuronal network physiology, will allow for greater development of treatment and prophylactic measures against epilepsy. The presence of peripheral inflammation, due to systemic diseases such as SLE or RA, has the potential capacity to damage the BBB and initiate or aggravate epileptogenesis. Therefore, the control of inflammation in such disorders may lessen the risk of developing epilepsy.

## Abbreviations

BBB: Blood–brain barrier
BD: Behcet’s disease
CCL2: Chemokine (C-C motif) ligand 2
CCR2: C-C chemokine receptor type 2
CD: Celiac Disease
CNS: Central nervous system
COX: Cyclooxygenase
CSF: Cerebrospinal fluid
CX3CL1: Chemokine (C-X3-C motif) ligand 1
CXCR4: C-X-C chemokine receptor type 4
GFAP: Glial fibrillary acidic protein
HS: Hippocampal sclerosis
IL: Interleukin
KO: Knock out
LPS: Lipopolysaccharide
LTP: Long-term potentiation
MFS: Mossy fiber sprouting
MIA: Maternal immune activation
MMP-9: Matrix metalloproteinase-9
MTLE: Mesial temporal lobe epilepsy
NSAID: Non-steroidal anti-inflammatory drug
PAF: Platelet activating factor
PG: Prostaglandin
PIC: Polyinosinic–polycytidylic acid
PTZ: Pentylenetetrazole
RA: Rheumatoid arthritis
SE: Status epilepticus
SLE: Systemic lupus erythematous
SS: Sjogren’s syndrome
TGF: Transforming growth factor
T1DM: Type I diabetes
TLE: Temporal lobe epilepsy
TLR: Toll-like receptors
TNF: Tumor necrosis factor
TRAIL: TNF-related apoptosis-inducing ligand
